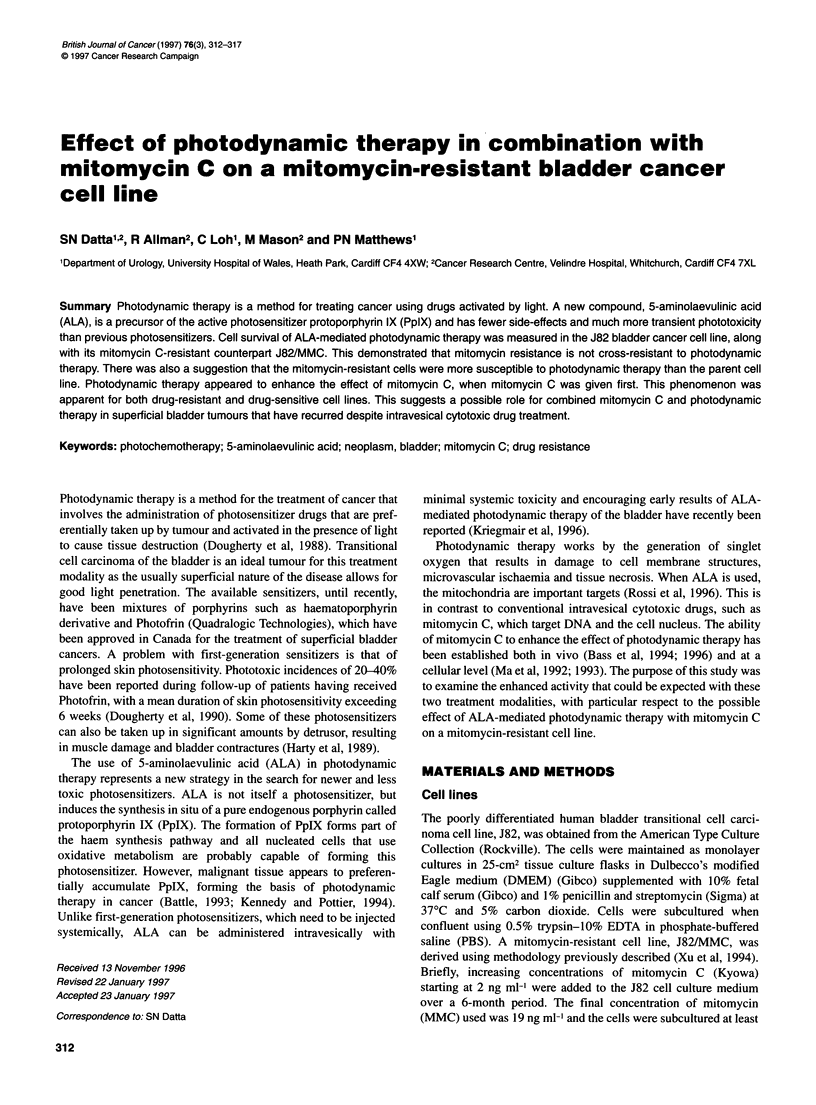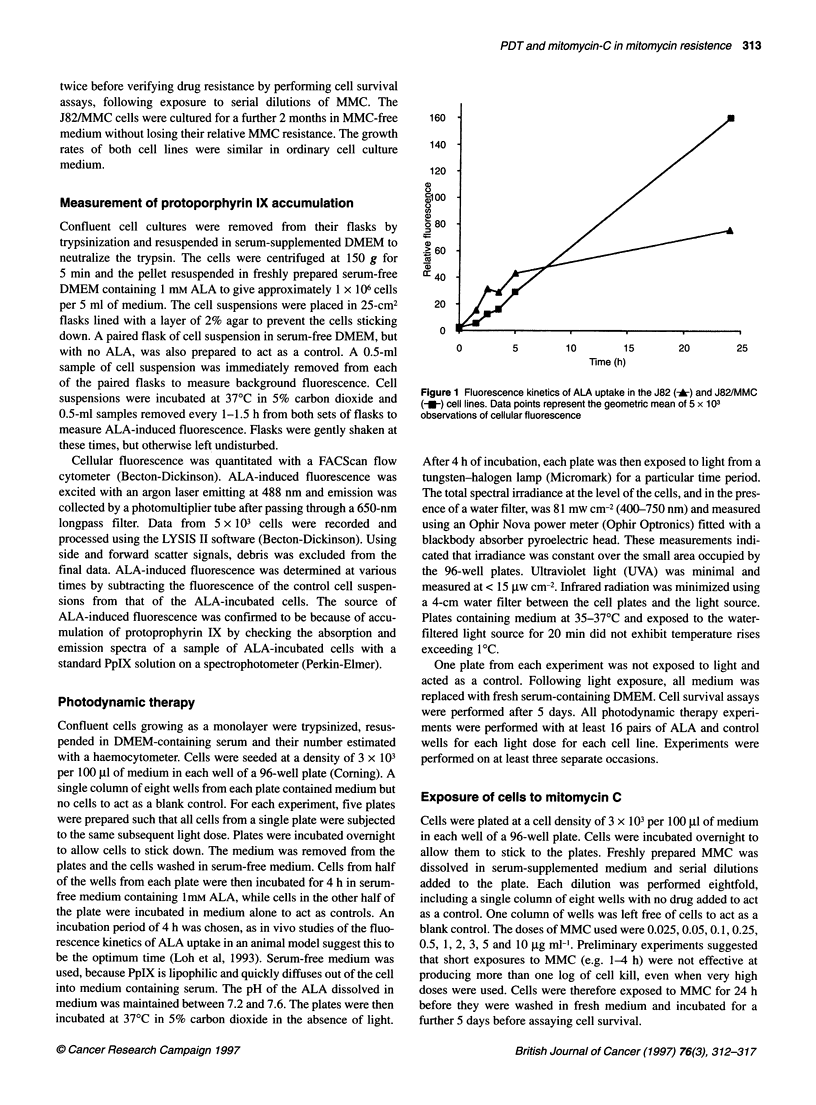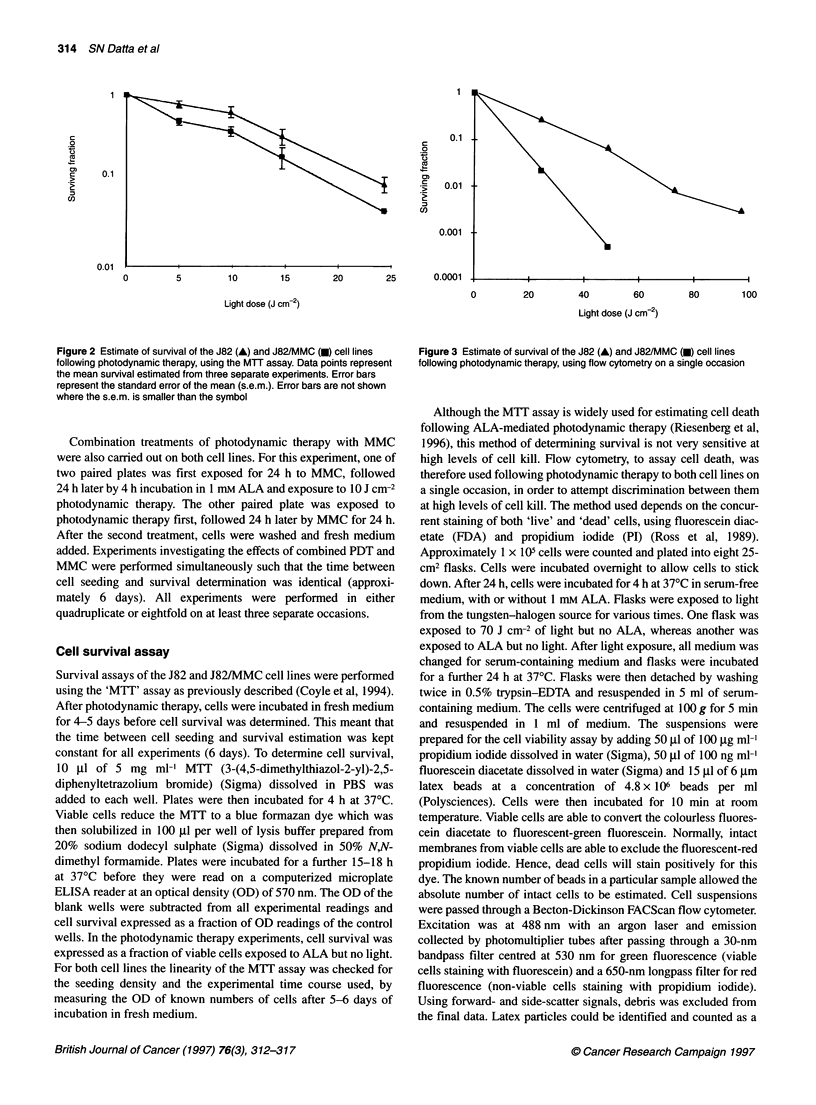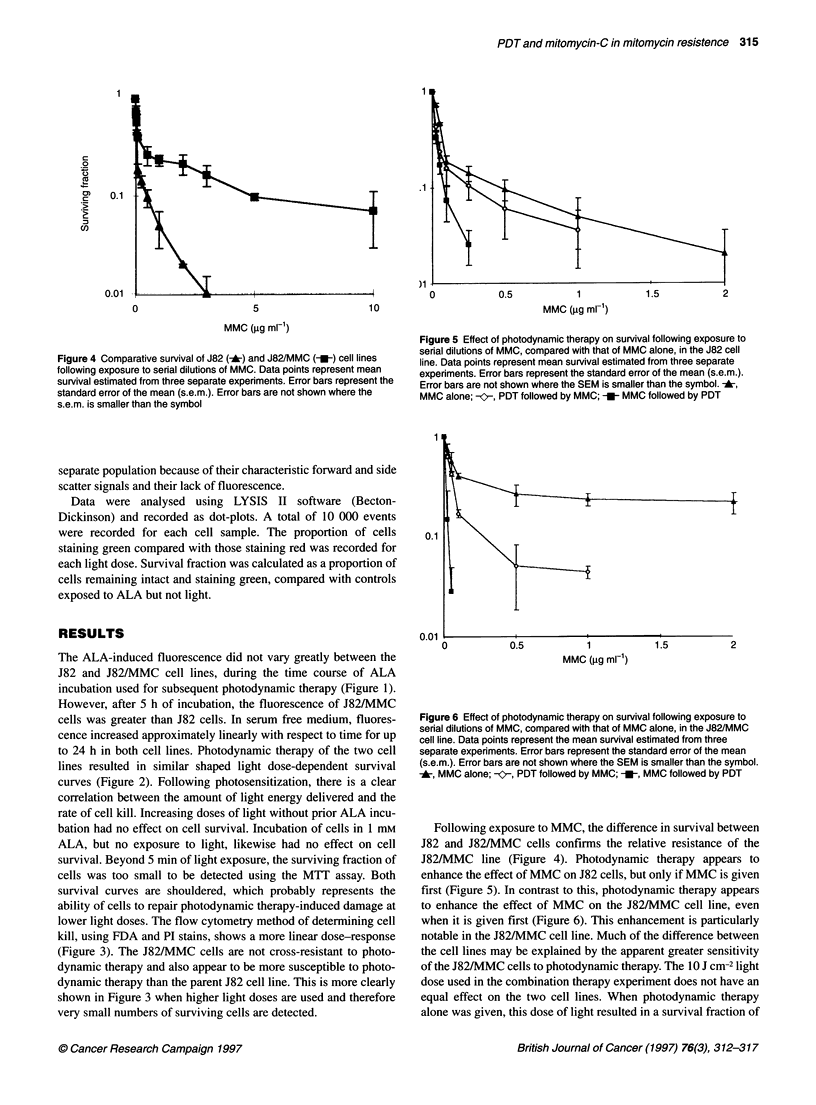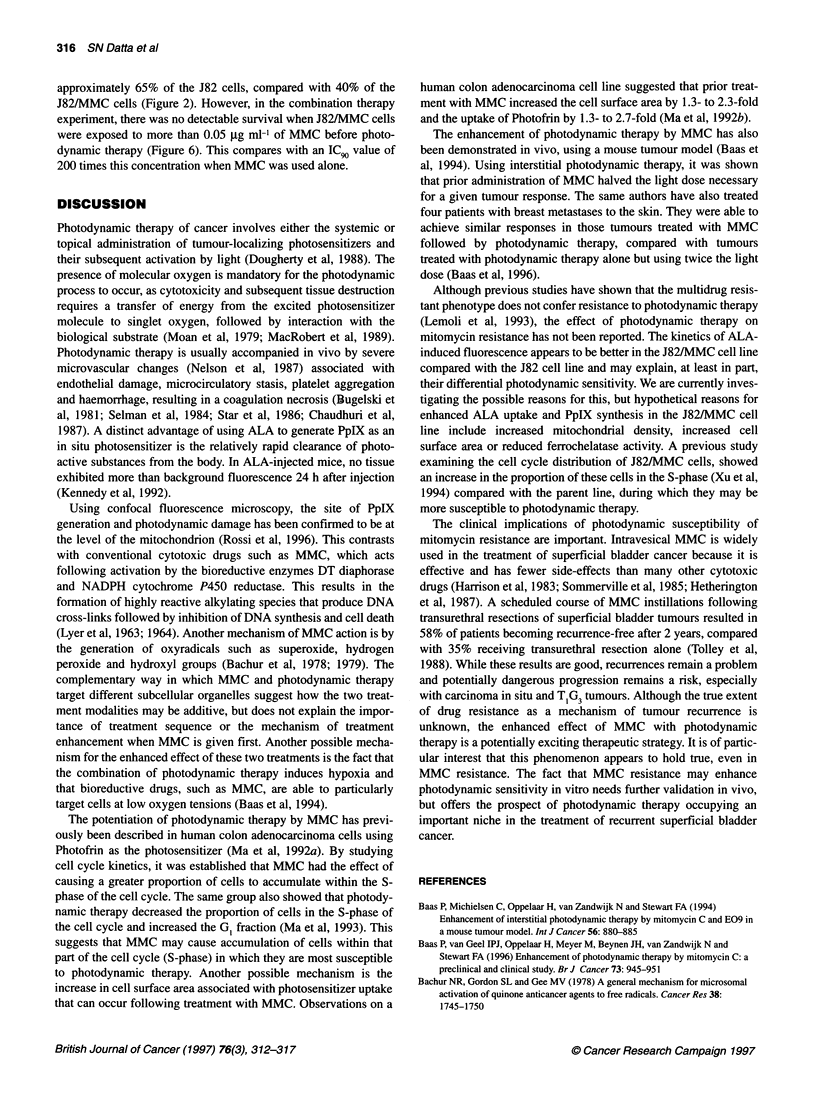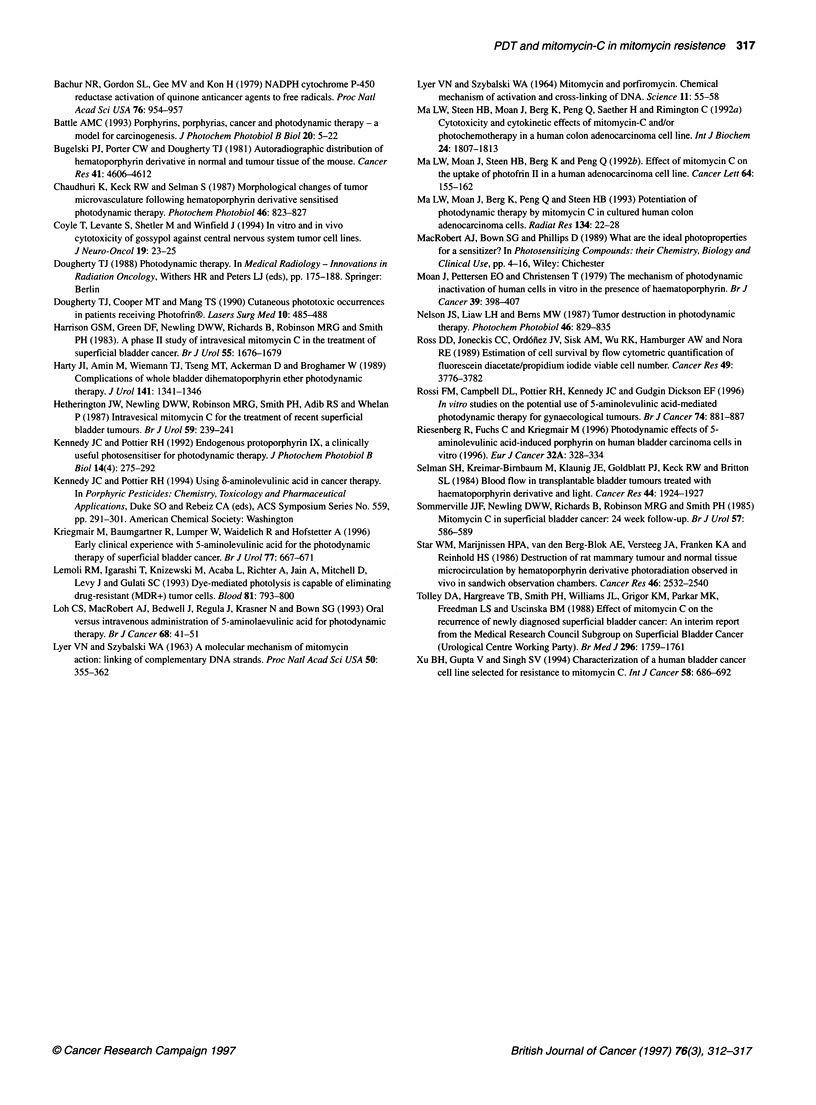# Effect of photodynamic therapy in combination with mitomycin C on a mitomycin-resistant bladder cancer cell line.

**DOI:** 10.1038/bjc.1997.384

**Published:** 1997

**Authors:** S. N. Datta, R. Allman, C. Loh, M. Mason, P. N. Matthews

**Affiliations:** Department of Urology, University Hospital of Wales, Heath Park, Cardiff.

## Abstract

Photodynamic therapy is a method for treating cancer using drugs activated by light. A new compound, 5-aminolaevulinic acid (ALA), is a precursor of the active photosensitizer protoporphyrin IX (PpIX) and has fewer side-effects and much more transient phototoxicity than previous photosensitizers. Cell survival of ALA-mediated photodynamic therapy was measured in the J82 bladder cancer cell line, along with its mitomycin C-resistant counterpart J82/MMC. This demonstrated that mitomycin resistance is not cross-resistant to photodynamic therapy. There was also a suggestion that the mitomycin-resistant cells were more susceptible to photodynamic therapy than the parent cell line. Photodynamic therapy appeared to enhance the effect of mitomycin C, when mitomycin C was given first. This phenomenon was apparent for both drug-resistant and drug-sensitive cell lines. This suggests a possible role for combined mitomycin C and photodynamic therapy in superficial bladder tumours that have recurred despite intravesical cytotoxic drug treatment.


British Journal of Cancer (1997) 76(3), 312–317
© 1997 Cancer Research Campaign

### Effect of photodynamic therapy in combination with mitomycin C on a mitomycin-resistant bladder cancer cell line

**SN Datta[1,2], R Allman[2], C Loh[1], M Mason[2] and PN Matthews[1]**

[1]Department of Urology, University Hospital of Wales, Heath Park, Cardiff CF4 4XW; [2]Cancer Research Centre, Velindre Hospital, Whitchurch, Cardiff CF4 7XL

**Summary** Photodynamic therapy is a method for treating cancer using drugs activated by light. A new compound, 5-aminolaevulinic acid (ALA), is a precursor of the active photosensitizer protoporphyrin IX (PpIX) and has fewer side-effects and much more transient phototoxicity than previous photosensitizers. Cell survival of ALA-mediated photodynamic therapy was measured in the J82 bladder cancer cell line, along with its mitomycin C-resistant counterpart J82/MMC. This demonstrated that mitomycin resistance is not cross-resistant to photodynamic therapy. There was also a suggestion that the mitomycin-resistant cells were more susceptible to photodynamic therapy than the parent cell line. Photodynamic therapy appeared to enhance the effect of mitomycin C, when mitomycin C was given first. This phenomenon was apparent for both drug-resistant and drug-sensitive cell lines. This suggests a possible role for combined mitomycin C and photodynamic therapy in superficial bladder tumours that have recurred despite intravesical cytotoxic drug treatment.

**Keywords:** photochemotherapy; 5-aminolaevulinic acid; neoplasm, bladder; mitomycin C; drug resistance

Photodynamic therapy is a method for the treatment of cancer that involves the administration of photosensitizer drugs that are preferentially taken up by tumour and activated in the presence of light to cause tissue destruction (Dougherty et al, 1988). Transitional cell carcinoma of the bladder is an ideal tumour for this treatment modality as the usually superficial nature of the disease allows for good light penetration. The available sensitizers, until recently, have been mixtures of porphyrins such as haematoporphyrin derivative and Photofrin (Quadralogic Technologies), which have been approved in Canada for the treatment of superficial bladder cancers. A problem with first-generation sensitizers is that of prolonged skin photosensitivity. Phototoxic incidences of 20–40% have been reported during follow-up of patients having received Photofrin, with a mean duration of skin photosensitivity exceeding 6 weeks (Dougherty et al, 1990). Some of these photosensitizers can also be taken up in significant amounts by detrusor, resulting in muscle damage and bladder contractures (Harty et al, 1989).

The use of 5-aminolaevulinic acid (ALA) in photodynamic therapy represents a new strategy in the search for newer and less toxic photosensitizers. ALA is not itself a photosensitizer, but induces the synthesis in situ of a pure endogenous porphyrin called protoporphyrin IX (PpIX). The formation of PpIX forms part of the haem synthesis pathway and all nucleated cells that use oxidative metabolism are probably capable of forming this photosensitizer. However, malignant tissue appears to preferentially accumulate PpIX, forming the basis of photodynamic therapy in cancer (Battle, 1993; Kennedy and Pottier, 1994). Unlike first-generation photosensitizers, which need to be injected systemically, ALA can be administered intravesically with minimal systemic toxicity and encouraging early results of ALA-mediated photodynamic therapy of the bladder have recently been reported (Kriegmair et al, 1996).

Photodynamic therapy works by the generation of singlet oxygen that results in damage to cell membrane structures, microvascular ischaemia and tissue necrosis. When ALA is used, the mitochondria are important targets (Rossi et al, 1996). This is in contrast to conventional intravesical cytotoxic drugs, such as mitomycin C, which target DNA and the cell nucleus. The ability of mitomycin C to enhance the effect of photodynamic therapy has been established both in vivo (Bass et al, 1994; 1996) and at a cellular level (Ma et al, 1992; 1993). The purpose of this study was to examine the enhanced activity that could be expected with these two treatment modalities, with particular respect to the possible effect of ALA-mediated photodynamic therapy with mitomycin C on a mitomycin-resistant cell line.

*Received 13 November 1996*
*Revised 22 January 1997*
*Accepted 23 January 1997*

*Correspondence to:* SN Datta

### MATERIALS AND METHODS

### Cell lines

The poorly differentiated human bladder transitional cell carcinoma cell line, J82, was obtained from the American Type Culture Collection (Rockville). The cells were maintained as monolayer cultures in 25-cm$^2$ tissue culture flasks in Dulbecco's modified Eagle medium (DMEM) (Gibco) supplemented with 10% fetal calf serum (Gibco) and 1% penicillin and streptomycin (Sigma) at 37°C and 5% carbon dioxide. Cells were subcultured when confluent using 0.5% trypsin–10% EDTA in phosphate-buffered saline (PBS). A mitomycin-resistant cell line, J82/MMC, was derived using methodology previously described (Xu et al, 1994). Briefly, increasing concentrations of mitomycin C (Kyowa) starting at 2 ng ml$^{-1}$ were added to the J82 cell culture medium over a 6-month period. The final concentration of mitomycin (MMC) used was 19 ng ml$^{-1}$ and the cells were subcultured at least twice before verifying drug resistance by performing cell survival assays, following exposure to serial dilutions of MMC. The J82/MMC cells were cultured for a further 2 months in MMC-free medium without losing their relative MMC resistance. The growth rates of both cell lines were similar in ordinary cell culture medium.

### Measurement of protoporphyrin IX accumulation

Confluent cell cultures were removed from their flasks by trypsinization and resuspended in serum-supplemented DMEM to neutralize the trypsin. The cells were centrifuged at 150 *g* for 5 min and the pellet resuspended in freshly prepared serum-free DMEM containing 1 mM ALA to give approximately $1 \times 10^6$ cells per 5 ml of medium. The cell suspensions were placed in 25-$cm^2$ flasks lined with a layer of 2% agar to prevent the cells sticking down. A paired flask of cell suspension in serum-free DMEM, but with no ALA, was also prepared to act as a control. A 0.5-ml sample of cell suspension was immediately removed from each of the paired flasks to measure background fluorescence. Cell suspensions were incubated at 37°C in 5% carbon dioxide and 0.5-ml samples removed every 1–1.5 h from both sets of flasks to measure ALA-induced fluorescence. Flasks were gently shaken at these times, but otherwise left undisturbed.

Cellular fluorescence was quantitated with a FACScan flow cytometer (Becton-Dickinson). ALA-induced fluorescence was excited with an argon laser emitting at 488 nm and emission was collected by a photomultiplier tube after passing through a 650-nm longpass filter. Data from $5 \times 10^3$ cells were recorded and processed using the LYSIS II software (Becton-Dickinson). Using side and forward scatter signals, debris was excluded from the final data. ALA-induced fluorescence was determined at various times by subtracting the fluorescence of the control cell suspensions from that of the ALA-incubated cells. The source of ALA-induced fluorescence was confirmed to be because of accumulation of protoprophyrin IX by checking the absorption and emission spectra of a sample of ALA-incubated cells with a standard PpIX solution on a spectrophotometer (Perkin-Elmer).

### Photodynamic therapy

Confluent cells growing as a monolayer were trypsinized, resuspended in DMEM-containing serum and their number estimated with a haemocytometer. Cells were seeded at a density of $3 \times 10^3$ per 100 µl of medium in each well of a 96-well plate (Corning). A single column of eight wells from each plate contained medium but no cells to act as a blank control. For each experiment, five plates were prepared such that all cells from a single plate were subjected to the same subsequent light dose. Plates were incubated overnight to allow cells to stick down. The medium was removed from the plates and the cells washed in serum-free medium. Cells from half of the wells from each plate were then incubated for 4 h in serum-free medium containing 1mM ALA, while cells in the other half of the plate were incubated in medium alone to act as controls. An incubation period of 4 h was chosen, as in vivo studies of the fluorescence kinetics of ALA uptake in an animal model suggest this to be the optimum time (Loh et al, 1993). Serum-free medium was used, because PpIX is lipophilic and quickly diffuses out of the cell into medium containing serum. The pH of the ALA dissolved in medium was maintained between 7.2 and 7.6. The plates were then incubated at 37°C in 5% carbon dioxide in the absence of light.

**Figure 1** Fluorescence kinetics of ALA uptake in the J82 (-▲-) and J82/MMC (-■-) cell lines. Data points represent the geometric mean of $5 \times 10^3$ observations of cellular fluorescence

After 4 h of incubation, each plate was then exposed to light from a tungsten–halogen lamp (Micromark) for a particular time period. The total spectral irradiance at the level of the cells, and in the presence of a water filter, was 81 mw $cm^{-2}$ (400–750 nm) and measured using an Ophir Nova power meter (Ophir Optronics) fitted with a blackbody absorber pyroelectric head. These measurements indicated that irradiance was constant over the small area occupied by the 96-well plates. Ultraviolet light (UVA) was minimal and measured at $< 15$ µw $cm^{-2}$. Infrared radiation was minimized using a 4-cm water filter between the cell plates and the light source. Plates containing medium at 35–37°C and exposed to the water-filtered light source for 20 min did not exhibit temperature rises exceeding 1°C.

One plate from each experiment was not exposed to light and acted as a control. Following light exposure, all medium was replaced with fresh serum-containing DMEM. Cell survival assays were performed after 5 days. All photodynamic therapy experiments were performed with at least 16 pairs of ALA and control wells for each light dose for each cell line. Experiments were performed on at least three separate occasions.

### Exposure of cells to mitomycin C

Cells were plated at a cell density of $3 \times 10^3$ per 100 µl of medium in each well of a 96-well plate. Cells were incubated overnight to allow them to stick to the plates. Freshly prepared MMC was dissolved in serum-supplemented medium and serial dilutions added to the plate. Each dilution was performed eightfold, including a single column of eight wells with no drug added to act as a control. One column of wells was left free of cells to act as a blank control. The doses of MMC used were 0.025, 0.05, 0.1, 0.25, 0.5, 1, 2, 3, 5 and 10 µg $ml^{-1}$. Preliminary experiments suggested that short exposures to MMC (e.g. 1–4 h) were not effective at producing more than one log of cell kill, even when very high doses were used. Cells were therefore exposed to MMC for 24 h before they were washed in fresh medium and incubated for a further 5 days before assaying cell survival.

© Cancer Research Campaign 1997

Figure 2 Estimate of survival of the J82 (▲) and J82/MMC (■) cell lines following photodynamic therapy, using the MTT assay. Data points represent the mean survival estimated from three separate experiments. Error bars represent the standard error of the mean (s.e.m.). Error bars are not shown where the s.e.m. is smaller than the symbol

Figure 3 Estimate of survival of the J82 (▲) and J82/MMC (■) cell lines following photodynamic therapy, using flow cytometry on a single occasion

Combination treatments of photodynamic therapy with MMC were also carried out on both cell lines. For this experiment, one of two paired plates was first exposed for 24 h to MMC, followed 24 h later by 4 h incubation in 1 mM ALA and exposure to 10 J cm$^{-2}$ photodynamic therapy. The other paired plate was exposed to photodynamic therapy first, followed 24 h later by MMC for 24 h. After the second treatment, cells were washed and fresh medium added. Experiments investigating the effects of combined PDT and MMC were performed simultaneously such that the time between cell seeding and survival determination was identical (approximately 6 days). All experiments were performed in either quadruplicate or eightfold on at least three separate occasions.

### Cell survival assay

Survival assays of the J82 and J82/MMC cell lines were performed using the 'MTT' assay as previously described (Coyle et al, 1994). After photodynamic therapy, cells were incubated in fresh medium for 4–5 days before cell survival was determined. This meant that the time between cell seeding and survival estimation was kept constant for all experiments (6 days). To determine cell survival, 10 µl of 5 mg ml$^{-1}$ MTT (3-(4,5-dimethylthiazol-2-yl)-2,5-diphenyltetrazolium bromide) (Sigma) dissolved in PBS was added to each well. Plates were then incubated for 4 h at 37°C. Viable cells reduce the MTT to a blue formazan dye which was then solubilized in 100 µl per well of lysis buffer prepared from 20% sodium dodecyl sulphate (Sigma) dissolved in 50% *N,N*-dimethyl formamide. Plates were incubated for a further 15–18 h at 37°C before they were read on a computerized microplate ELISA reader at an optical density (OD) of 570 nm. The OD of the blank wells were subtracted from all experimental readings and cell survival expressed as a fraction of OD readings of the control wells. In the photodynamic therapy experiments, cell survival was expressed as a fraction of viable cells exposed to ALA but no light. For both cell lines the linearity of the MTT assay was checked for the seeding density and the experimental time course used, by measuring the OD of known numbers of cells after 5–6 days of incubation in fresh medium.

Although the MTT assay is widely used for estimating cell death following ALA-mediated photodynamic therapy (Riesenberg et al, 1996), this method of determining survival is not very sensitive at high levels of cell kill. Flow cytometry, to assay cell death, was therefore used following photodynamic therapy to both cell lines on a single occasion, in order to attempt discrimination between them at high levels of cell kill. The method used depends on the concurrent staining of both 'live' and 'dead' cells, using fluorescein diacetate (FDA) and propidium iodide (PI) (Ross et al, 1989). Approximately $1 \times 10^5$ cells were counted and plated into eight 25-cm$^2$ flasks. Cells were incubated overnight to allow cells to stick down. After 24 h, cells were incubated for 4 h at 37°C in serum-free medium, with or without 1 mM ALA. Flasks were exposed to light from the tungsten–halogen source for various times. One flask was exposed to 70 J cm$^{-2}$ of light but no ALA, whereas another was exposed to ALA but no light. After light exposure, all medium was changed for serum-containing medium and flasks were incubated for a further 24 h at 37°C. Flasks were then detached by washing twice in 0.5% trypsin–EDTA and resuspended in 5 ml of serum-containing medium. The cells were centrifuged at 100 *g* for 5 min and resuspended in 1 ml of medium. The suspensions were prepared for the cell viability assay by adding 50 µl of 100 µg ml$^{-1}$ propidium iodide dissolved in water (Sigma), 50 µl of 100 ng ml$^{-1}$ fluorescein diacetate dissolved in water (Sigma) and 15 µl of 6 µm latex beads at a concentration of $4.8 \times 10^6$ beads per ml (Polysciences). Cells were then incubated for 10 min at room temperature. Viable cells are able to convert the colourless fluorescein diacetate to fluorescent-green fluorescein. Normally, intact membranes from viable cells are able to exclude the fluorescent-red propidium iodide. Hence, dead cells will stain positively for this dye. The known number of beads in a particular sample allowed the absolute number of intact cells to be estimated. Cell suspensions were passed through a Becton-Dickinson FACScan flow cytometer. Excitation was at 488 nm with an argon laser and emission collected by photomultiplier tubes after passing through a 30-nm bandpass filter centred at 530 nm for green fluorescence (viable cells staining with fluorescein) and a 650-nm longpass filter for red fluorescence (non-viable cells staining with propidium iodide). Using forward- and side-scatter signals, debris was excluded from the final data. Latex particles could be identified and counted as a

© Cancer Research Campaign 1997

**Figure 4** Comparative survival of J82 (▲) and J82/MMC (■) cell lines following exposure to serial dilutions of MMC. Data points represent mean survival estimated from three separate experiments. Error bars represent the standard error of the mean (s.e.m.). Error bars are not shown where the s.e.m. is smaller than the symbol

**Figure 5** Effect of photodynamic therapy on survival following exposure to serial dilutions of MMC, compared with that of MMC alone, in the J82 cell line. Data points represent mean survival estimated from three separate experiments. Error bars represent the standard error of the mean (s.e.m.). Error bars are not shown where the SEM is smaller than the symbol. ▲, MMC alone; ◇, PDT followed by MMC; ■ MMC followed by PDT

separate population because of their characteristic forward and side scatter signals and their lack of fluorescence.

Data were analysed using LYSIS II software (Becton-Dickinson) and recorded as dot-plots. A total of 10 000 events were recorded for each cell sample. The proportion of cells staining green compared with those staining red was recorded for each light dose. Survival fraction was calculated as a proportion of cells remaining intact and staining green, compared with controls exposed to ALA but not light.

### RESULTS

The ALA-induced fluorescence did not vary greatly between the J82 and J82/MMC cell lines, during the time course of ALA incubation used for subsequent photodynamic therapy (Figure 1). However, after 5 h of incubation, the fluorescence of J82/MMC cells was greater than J82 cells. In serum free medium, fluorescence increased approximately linearly with respect to time for up to 24 h in both cell lines. Photodynamic therapy of the two cell lines resulted in similar shaped light dose-dependent survival curves (Figure 2). Following photosensitization, there is a clear correlation between the amount of light energy delivered and the rate of cell kill. Increasing doses of light without prior ALA incubation had no effect on cell survival. Incubation of cells in 1 mM ALA, but no exposure to light, likewise had no effect on cell survival. Beyond 5 min of light exposure, the surviving fraction of cells was too small to be detected using the MTT assay. Both survival curves are shouldered, which probably represents the ability of cells to repair photodynamic therapy-induced damage at lower light doses. The flow cytometry method of determining cell kill, using FDA and PI stains, shows a more linear dose–response (Figure 3). The J82/MMC cells are not cross-resistant to photodynamic therapy and also appear to be more susceptible to photodynamic therapy than the parent J82 cell line. This is more clearly shown in Figure 3 when higher light doses are used and therefore very small numbers of surviving cells are detected.

**Figure 6** Effect of photodynamic therapy on survival following exposure to serial dilutions of MMC, compared with that of MMC alone, in the J82/MMC cell line. Data points represent the mean survival estimated from three separate experiments. Error bars represent the standard error of the mean (s.e.m.). Error bars are not shown where the SEM is smaller than the symbol. ▲, MMC alone; ◇, PDT followed by MMC; ■, MMC followed by PDT

Following exposure to MMC, the difference in survival between J82 and J82/MMC cells confirms the relative resistance of the J82/MMC line (Figure 4). Photodynamic therapy appears to enhance the effect of MMC on J82 cells, but only if MMC is given first (Figure 5). In contrast to this, photodynamic therapy appears to enhance the effect of MMC on the J82/MMC cell line, even when it is given first (Figure 6). This enhancement is particularly notable in the J82/MMC cell line. Much of the difference between the cell lines may be explained by the apparent greater sensitivity of the J82/MMC cells to photodynamic therapy. The 10 J $cm^{-2}$ light dose used in the combination therapy experiment does not have an equal effect on the two cell lines. When photodynamic therapy alone was given, this dose of light resulted in a survival fraction of

© Cancer Research Campaign 1997 

approximately 65% of the J82 cells, compared with 40% of the J82/MMC cells (Figure 2). However, in the combination therapy experiment, there was no detectable survival when J82/MMC cells were exposed to more than 0.05 μg ml$^{-1}$ of MMC before photodynamic therapy (Figure 6). This compares with an $IC_{90}$ value of 200 times this concentration when MMC was used alone.

### DISCUSSION

Photodynamic therapy of cancer involves either the systemic or topical administration of tumour-localizing photosensitizers and their subsequent activation by light (Dougherty et al, 1988). The presence of molecular oxygen is mandatory for the photodynamic process to occur, as cytotoxicity and subsequent tissue destruction requires a transfer of energy from the excited photosensitizer molecule to singlet oxygen, followed by interaction with the biological substrate (Moan et al, 1979; MacRobert et al, 1989). Photodynamic therapy is usually accompanied in vivo by severe microvascular changes (Nelson et al, 1987) associated with endothelial damage, microcirculatory stasis, platelet aggregation and haemorrhage, resulting in a coagulation necrosis (Bugelski et al, 1981; Selman et al, 1984; Star et al, 1986; Chaudhuri et al, 1987). A distinct advantage of using ALA to generate PpIX as an in situ photosensitizer is the relatively rapid clearance of photoactive substances from the body. In ALA-injected mice, no tissue exhibited more than background fluorescence 24 h after injection (Kennedy et al, 1992).

Using confocal fluorescence microscopy, the site of PpIX generation and photodynamic damage has been confirmed to be at the level of the mitochondrion (Rossi et al, 1996). This contrasts with conventional cytotoxic drugs such as MMC, which acts following activation by the bioreductive enzymes DT diaphorase and NADPH cytochrome *P*450 reductase. This results in the formation of highly reactive alkylating species that produce DNA cross-links followed by inhibition of DNA synthesis and cell death (Lyer et al, 1963; 1964). Another mechanism of MMC action is by the generation of oxyradicals such as superoxide, hydrogen peroxide and hydroxyl groups (Bachur et al, 1978; 1979). The complementary way in which MMC and photodynamic therapy target different subcellular organelles suggest how the two treatment modalities may be additive, but does not explain the importance of treatment sequence or the mechanism of treatment enhancement when MMC is given first. Another possible mechanism for the enhanced effect of these two treatments is the fact that the combination of photodynamic therapy induces hypoxia and that bioreductive drugs, such as MMC, are able to particularly target cells at low oxygen tensions (Baas et al, 1994).

The potentiation of photodynamic therapy by MMC has previously been described in human colon adenocarcinoma cells using Photofrin as the photosensitizer (Ma et al, 1992*a*). By studying cell cycle kinetics, it was established that MMC had the effect of causing a greater proportion of cells to accumulate within the S-phase of the cell cycle. The same group also showed that photodynamic therapy decreased the proportion of cells in the S-phase of the cell cycle and increased the $G_1$ fraction (Ma et al, 1993). This suggests that MMC may cause accumulation of cells within that part of the cell cycle (S-phase) in which they are most susceptible to photodynamic therapy. Another possible mechanism is the increase in cell surface area associated with photosensitizer uptake that can occur following treatment with MMC. Observations on a human colon adenocarcinoma cell line suggested that prior treatment with MMC increased the cell surface area by 1.3- to 2.3-fold and the uptake of Photofrin by 1.3- to 2.7-fold (Ma et al, 1992*b*).

The enhancement of photodynamic therapy by MMC has also been demonstrated in vivo, using a mouse tumour model (Baas et al, 1994). Using interstitial photodynamic therapy, it was shown that prior administration of MMC halved the light dose necessary for a given tumour response. The same authors have also treated four patients with breast metastases to the skin. They were able to achieve similar responses in those tumours treated with MMC followed by photodynamic therapy, compared with tumours treated with photodynamic therapy alone but using twice the light dose (Baas et al, 1996).

Although previous studies have shown that the multidrug resistant phenotype does not confer resistance to photodynamic therapy (Lemoli et al, 1993), the effect of photodynamic therapy on mitomycin resistance has not been reported. The kinetics of ALA-induced fluorescence appears to be better in the J82/MMC cell line compared with the J82 cell line and may explain, at least in part, their differential photodynamic sensitivity. We are currently investigating the possible reasons for this, but hypothetical reasons for enhanced ALA uptake and PpIX synthesis in the J82/MMC cell line include increased mitochondrial density, increased cell surface area or reduced ferrochelatase activity. A previous study examining the cell cycle distribution of J82/MMC cells, showed an increase in the proportion of these cells in the S-phase (Xu et al, 1994) compared with the parent line, during which they may be more susceptible to photodynamic therapy.

The clinical implications of photodynamic susceptibility of mitomycin resistance are important. Intravesical MMC is widely used in the treatment of superficial bladder cancer because it is effective and has fewer side-effects than many other cytotoxic drugs (Harrison et al, 1983; Sommerville et al, 1985; Hetherington et al, 1987). A scheduled course of MMC instillations following transurethral resections of superficial bladder tumours resulted in 58% of patients becoming recurrence-free after 2 years, compared with 35% receiving transurethral resection alone (Tolley et al, 1988). While these results are good, recurrences remain a problem and potentially dangerous progression remains a risk, especially with carcinoma in situ and $T_1G_3$ tumours. Although the true extent of drug resistance as a mechanism of tumour recurrence is unknown, the enhanced effect of MMC with photodynamic therapy is a potentially exciting therapeutic strategy. It is of particular interest that this phenomenon appears to hold true, even in MMC resistance. The fact that MMC resistance may enhance photodynamic sensitivity in vitro needs further validation in vivo, but offers the prospect of photodynamic therapy occupying an important niche in the treatment of recurrent superficial bladder cancer.

### REFERENCES

Baas P, Michielsen C, Oppelaar H, van Zandwijk N and Stewart FA (1994) Enhancement of interstitial photodynamic therapy by mitomycin C and EO9 in a mouse tumour model. *Int J Cancer* **56**: 880–885

Baas P, van Geel IPJ, Oppelaar H, Meyer M, Beynen JH, van Zandwijk N and Stewart FA (1996) Enhancement of photodynamic therapy by mitomycin C: a preclinical and clinical study. *Br J Cancer* **73**: 945–951

Bachur NR, Gordon SL and Gee MV (1978) A general mechanism for microsomal activation of quinone anticancer agents to free radicals. *Cancer Res* **38**: 1745–1750

© Cancer Research Campaign 1997

Bachur NR, Gordon SL, Gee MV and Kon H (1979) NADPH cytochrome P-450 reductase activation of quinone anticancer agents to free radicals. *Proc Natl Acad Sci USA* **76**: 954–957

Battle AMC (1993) Porphyrins, porphyrias, cancer and photodynamic therapy – a model for carcinogenesis. *J Photochem Photobiol B Biol* **20**: 5–22

Bugelski PJ, Porter CW and Dougherty TJ (1981) Autoradiographic distribution of hematoporphyrin derivative in normal and tumour tissue of the mouse. *Cancer Res* **41**: 4606–4612

Chaudhuri K, Keck RW and Selman S (1987) Morphological changes of tumor microvasculature following hematoporphyrin derivative sensitised photodynamic therapy. *Photochem Photobiol* **46**: 823–827

Coyle T, Levante S, Shetler M and Winfield J (1994) In vitro and in vivo cytotoxicity of gossypol against central nervous system tumor cell lines. *J Neuro-Oncol* **19**: 23–25

Dougherty TJ (1988) Photodynamic therapy. In *Medical Radiology – Innovations in Radiation Oncology*, Withers HR and Peters LJ (eds), pp. 175–188. Springer: Berlin

Dougherty TJ, Cooper MT and Mang TS (1990) Cutaneous phototoxic occurrences in patients receiving Photofrin®. *Lasers Surg Med* **10**: 485–488

Harrison GSM, Green DF, Newling DWW, Richards B, Robinson MRG and Smith PH (1983). A phase II study of intravesical mitomycin C in the treatment of superficial bladder cancer. *Br J Urol* **55**: 1676–1679

Harty JI, Amin M, Wiemann TJ, Tseng MT, Ackerman D and Broghamer W (1989) Complications of whole bladder dihematoporphyrin ether photodynamic therapy. *J Urol* **141**: 1341–1346

Hetherington JW, Newling DWW, Robinson MRG, Smith PH, Adib RS and Whelan P (1987) Intravesical mitomycin C for the treatment of recent superficial bladder tumours. *Br J Urol* **59**: 239–241

Kennedy JC and Pottier RH (1992) Endogenous protoporphyrin IX, a clinically useful photosensitiser for photodynamic therapy. *J Photochem Photobiol B Biol* **14**(4): 275–292

Kennedy JC and Pottier RH (1994) Using δ-aminolevulinic acid in cancer therapy. In *Porphyric Pesticides: Chemistry, Toxicology and Pharmaceutical Applications*, Duke SO and Rebeiz CA (eds), ACS Symposium Series No. 559, pp. 291–301. American Chemical Society: Washington

Kriegmair M, Baumgartner R, Lumper W, Waidelich R and Hofstetter A (1996) Early clinical experience with 5-aminolevulinic acid for the photodynamic therapy of superficial bladder cancer. *Br J Urol* **77**: 667–671

Lemoli RM, Igarashi T, Knizewski M, Acaba L, Richter A, Jain A, Mitchell D, Levy J and Gulati SC (1993) Dye-mediated photolysis is capable of eliminating drug-resistant (MDR+) tumor cells. *Blood* **81**: 793–800

Loh CS, MacRobert AJ, Bedwell J, Regula J, Krasner N and Bown SG (1993) Oral versus intravenous administration of 5-aminolaevulinic acid for photodynamic therapy. *Br J Cancer* **68**: 41–51

Lyer VN and Szybalski WA (1963) A molecular mechanism of mitomycin action: linking of complementary DNA strands. *Proc Natl Acad Sci USA* **50**: 355–362

Lyer VN and Szybalski WA (1964) Mitomycin and porfiromycin. Chemical mechanism of activation and cross-linking of DNA. *Science* **11**: 55–58

Ma LW, Steen HB, Moan J, Berg K, Peng Q, Saether H and Rimington C (1992*a*) Cytotoxicity and cytokinetic effects of mitomycin-C and/or photochemotherapy in a human colon adenocarcinoma cell line. *Int J Biochem* **24**: 1807–1813

Ma LW, Moan J, Steen HB, Berg K and Peng Q (1992*b*). Effect of mitomycin C on the uptake of photofrin II in a human adenocarcinoma cell line. *Cancer Lett* **64**: 155–162

Ma LW, Moan J, Berg K, Peng Q and Steen HB (1993) Potentiation of photodynamic therapy by mitomycin C in cultured human colon adenocarcinoma cells. *Radiat Res* **134**: 22–28

MacRobert AJ, Bown SG and Phillips D (1989) What are the ideal photoproperties for a sensitizer? In *Photosensitizing Compounds: their Chemistry, Biology and Clinical Use*, pp. 4–16, Wiley: Chichester

Moan J, Pettersen EO and Christensen T (1979) The mechanism of photodynamic inactivation of human cells in vitro in the presence of haematoporphyrin. *Br J Cancer* **39**: 398–407

Nelson JS, Liaw LH and Berns MW (1987) Tumor destruction in photodynamic therapy. *Photochem Photobiol* **46**: 829–835

Ross DD, Joneckis CC, Ordóñez JV, Sisk AM, Wu RK, Hamburger AW and Nora RE (1989) Estimation of cell survival by flow cytometric quantification of fluorescein diacetate/propidium iodide viable cell number. *Cancer Res* **49**: 3776–3782

Rossi FM, Campbell DL, Pottier RH, Kennedy JC and Gudgin Dickson EF (1996) *In vitro* studies on the potential use of 5-aminolevulinic acid-mediated photodynamic therapy for gynaecological tumours. *Br J Cancer* **74**: 881–887

Riesenberg R, Fuchs C and Kriegmair M (1996) Photodynamic effects of 5-aminolevulinic acid-induced porphyrin on human bladder carcinoma cells in vitro (1996). *Eur J Cancer* **32A**: 328–334

Selman SH, Kreimar-Birnbaum M, Klaunig JE, Goldblatt PJ, Keck RW and Britton SL (1984) Blood flow in transplantable bladder tumours treated with haematoporphyrin derivative and light. *Cancer Res* **44**: 1924–1927

Sommerville JJF, Newling DWW, Richards B, Robinson MRG and Smith PH (1985) Mitomycin C in superficial bladder cancer: 24 week follow-up. *Br J Urol* **57**: 586–589

Star WM, Marijnissen HPA, van den Berg-Blok AE, Versteeg JA, Franken KA and Reinhold HS (1986) Destruction of rat mammary tumour and normal tissue microcirculation by hematoporphyrin derivative photoradiation observed in vivo in sandwich observation chambers. *Cancer Res* **46**: 2532–2540

Tolley DA, Hargreave TB, Smith PH, Williams JL, Grigor KM, Parkar MK, Freedman LS and Uscinska BM (1988) Effect of mitomycin C on the recurrence of newly diagnosed superficial bladder cancer: An interim report from the Medical Research Council Subgroup on Superficial Bladder Cancer (Urological Centre Working Party). *Br Med J* **296**: 1759–1761

Xu BH, Gupta V and Singh SV (1994) Characterization of a human bladder cancer cell line selected for resistance to mitomycin C. *Int J Cancer* **58**: 686–692

© Cancer Research Campaign 1997